# Maternal Snoring May Predict Adverse Pregnancy Outcomes: A Cohort Study in China

**DOI:** 10.1371/journal.pone.0148732

**Published:** 2016-02-12

**Authors:** Xing Ge, Fangbiao Tao, Kun Huang, Leijing Mao, Sanhuan Huang, Ying Niu, Jiahu Hao, Yanli Sun, Erigene Rutayisire

**Affiliations:** 1 Department of Maternal and Child Health, School of Public Health, Anhui Medical University, 81 Meishan Road, Hefei, 230032, Anhui, China; 2 Anhui Provincial Key Laboratory of Population Health & Aristogenics, 81 Meishan Road, Hefei, 230032, Anhui, China; Weill Cornell Medical College in Qatar, QATAR

## Abstract

**Objective:**

To examine the prevalence of snoring during pregnancy and its effects on key pregnancy outcomes.

**Methods:**

Pregnant women were consecutively recruited in their first trimester. Habitual snoring was screened by using a questionnaire in the 1^st^ and 3^rd^ trimester, respectively. According to the time of snoring, participants were divided into pregnancy onset snorers, chronic snorers and non-snorers. Logistic regressions were performed to examine the associations between snoring and pregnancy outcomes.

**Results:**

Of 3 079 pregnant women, 16.6% were habitual snorers, with 11.7% were pregnancy onset snorers and 4.9% were chronic snorers. After adjusting for potential confounders, chronic snorers were independently associated with gestational diabetes mellitus (GDM) (*RR* 1.66, 95%*CI* 1.09–2.53). Both pregnancy onset and chronic snorers were independently associated with placental adhesion (*RR* 1.96, 95%*CI* 1.17–3.27, and *RR* 2.33, 95%*CI* 1.22–4.46, respectively). Pregnancy onset snorers were at higher risk of caesarean delivery (*RR* 1.37, 95%*CI* 1.09–1.73) and having macrosomia (*RR* 1.54, 95%*CI* 1.05–2.27) and large for gestational age (LGA) (*RR* 1.71, 95%*CI* 1.31–2.24) infants. In addition, being overweight or obese before pregnancy plays an important role in mediating snoring and adverse pregnancy outcomes.

**Conclusions:**

Maternal snoring may increase the risk of adverse pregnancy outcomes, and being overweight or obese before pregnancy with snoring is remarkable for researchers. Further studies are still needed to confirm our results.

## Introduction

Sleep-disordered breathing (SDB) is prevalent among adults, and now it is considered as a public health problem worldwide [1]. Mounting evidence shows the frequency of SDB is much higher in pregnant women compared to non-pregnant women [2–6]. Snoring is reported as one of the most common symptoms observed among people with SDB [7], and increasing of upper airway resistance and obstructive sleep apnea (OSA) are regarded as SDB characteristics [8]. Pregnancy physiology predisposes women to the development of airflow limitations during sleep, and sleep-disordered breathing such as snoring and OSA increases the risk of airflow limitations. Therefore, pregnant women with snoring or OSA were more likely to have airflow limitations than non-pregnant population [9,10].

Pregnancy is a critical period for women of childbearing age, where known or unknown factors can affect maternal-fetal health. Studies demonstrated that snoring habit was associated with adverse pregnancy outcomes, including GDM, gestational hypertension, preeclampsia, cesarean delivery [11–15], preterm birth, low birth weight (LBW), small for gestational age (SGA) [16–21] and so on. However, conflicting results also reported in many studies. A prospective study including 105 pregnant women found no relationship between SDB and adverse pregnancy outcomes [22]. Therefore, Redhead K et al doubted whether SDB can really cause adverse pregnancy outcomes [23].

The potential mechanism of maternal snoring on maternal-fetal health is likely complicated. There were studies that indicated that overweight and obese pregnant women were at higher risk of SDB than their lean weight counterparts [24,25]. It suggested that being overweight or obese before pregnancy may play an important role in developing habitual snoring and result in adverse maternal-fetal outcomes. Hence, further studies are needed to consistently investigate the relationship between maternal snoring and pregnancy outcomes. The objective of the present study was to determine the prevalence of snoring during pregnancy, as well as to examine the effect of maternal snoring on key pregnancy outcomes.

## Materials and Methods

This study was based on Ma’anshan Birth Cohort Study (MABC). The cohort was designed to investigate the effects of prenatal exposure on adverse pregnancy outcomes, child health and development in China. Pregnant women were consecutively recruited from antenatal clinics of Maternal and Child Health (MCH) Care Centre in Ma’anshan, Anhui Province of China from May 2013 to September 2014 by trained investigators. The centre contains about eighty percent of all pregnant women in Ma’anshan. Pregnant women were invited to participate in at their first visit to the centre, and women≥18 years old and≤14 weeks pregnant with a singleton pregnancy were eligible. Gestational age and date of delivery were calculated using last menstrual period data or ultrasound to estimate if their menstruation were irregular [26]. Women with serious liver or kidney disease, serious cerebrovascular disease, serious mental disease, mental retardation, or unable to complete the study independently were excluded. The study was approved by the ethical committee of Anhui Medical University. Written informed consent was obtained from all participants.

To evaluate snoring status of pregnant women, in the first and third trimester of pregnancy at the time of their routine obstetric visit, they were asked to complete a questionnaire, respectively. In the first questionnaire, pregnant women were asked to report the frequency with which they experienced snoring just before pregnancy, the second questionnaire evaluated whether or not they snored during pregnancy, reported in terms of “never”, “1–2 times per month”, “1–2 times per week”, “3–4 times per week” or “almost every day”. Similarly, the two questionnaires investigated whether they had symptoms of witnessed apneas before and during pregnancy. We also asked if their bed partners had complained about their snoring.

Pregnant women were divided into three groups according to how their snoring developed. Habitual snorers were regarded as women who snored at least 3–4 times per week. Pregnancy onset snorers were defined as women whose habitual snoring started during pregnancy, while chronic snorers were women who snored both before and during pregnancy. Women who reported that they snored “never”, “1–2 times per month” or “1–2 times per week”, were considered as non-snorers. Likewise, witnessed apneas were defined as pregnant women had witnessed apneas at least 3–4 times per week.

Pre-pregnancy BMI was calculated from self-reported weight and height measured before conception, and we validated the data from their first visit to the centre by a trained investigator. Pre-pregnancy BMI was categorized according to the standard of Working Group on Obesity in China [27], as follows: underweight (BMI<18.5kg/m^2^), normal weight (18.5≤BMI<24.0 kg/m^2^), overweight (24.0≤BMI<28.0 kg/m^2^), and obesity (BMI≥28.0kg/m^2^). Diagnosis of GDM, gestational hypertension, and preeclampsia were obtained from medical records, as defined according to the American Diabetes Association (ADA) for gestational diabetes [28], gestational hypertension was defined as systolic pressure≥140 mmHg or diastolic pressure≥90 mmHg after gestation, and preeclampsia was systolic pressure≥140 mmHg or diastolic pressure≥90 mmHg with proteinuria≥0.3g in 24h or random urine protein≥ +after 20 weeks of gestation. Results of placental adhesion were obtained from delivery records. It was diagnosed by obstetrician or midwives and histologic confirmation. Placenta adhesion was defined as the presence of one of the four criteria according to Hung etc [29]: (1) difficult manual and piecemeal removal of placenta if there is no placenta separation 20 minutes after delivery, despite active management of the third stage of labor, according to written hospital protocols; (2) ultrasonic examination shows there are retained placental fragments that requiring curettage after a vaginal delivery; (3) massive bleeding from the parts of placental attachment after placenta removal during cesarean, managed conservatively dealing with resection of part of the attached placenta and the uterine wall, or stitching bleeding part; and (4) histologic evidence of a hysterectomy specimen. Other key variables regarding maternal demographics and neonatal outcomes such as mode of delivery, gestational age, and birth weight were obtained from medical records. SGA and LGA were calculated according to the latest standard [30]. Preterm birth was defined as born before 37 gestational weeks. Birth weight less than 2500g was considered as LBW, more than 4000g was regarded as macrosomia.

## Statistical Analysis

Assuming a frequency of 5.0% in gestational hypertension group and 95.0% in normal group, and assuming as an alpha of 0.05, the required sample size was estimated to be 2 048 according to the formula for calculating a sample size of a cohort study before the study began. In this study, with a sample of 3 474, we have 92.65% power to detect this difference.

All data collected were double entered into a database to assure data accuracy. In this study, characteristics of participants were shown as mean and standard deviation (SD) for continuous variables, and percentage for discrete variables. Between groups of comparisons were performed by using one-way analysis of variance (ANOVA) (pregnancy onset snorers, chronic snorers and non-snorers) for continuous variables, categorical variables were compared with *χ*^*2*^ test. Logistic regressions were used to assess the associations between snoring and pregnancy outcomes. Variables as pre-pregnancy BMI, maternal age, educational level, gravidity, only child or not and maternal smoking were adjusted as confounders. *RR*s and 95% confidence interval (*CI*) were calculated. All data were analyzed by using SPSS version 10.0. A two-tailed P-value less than 0.05 was considered statistically significant.

## Results

**Fig 1:**
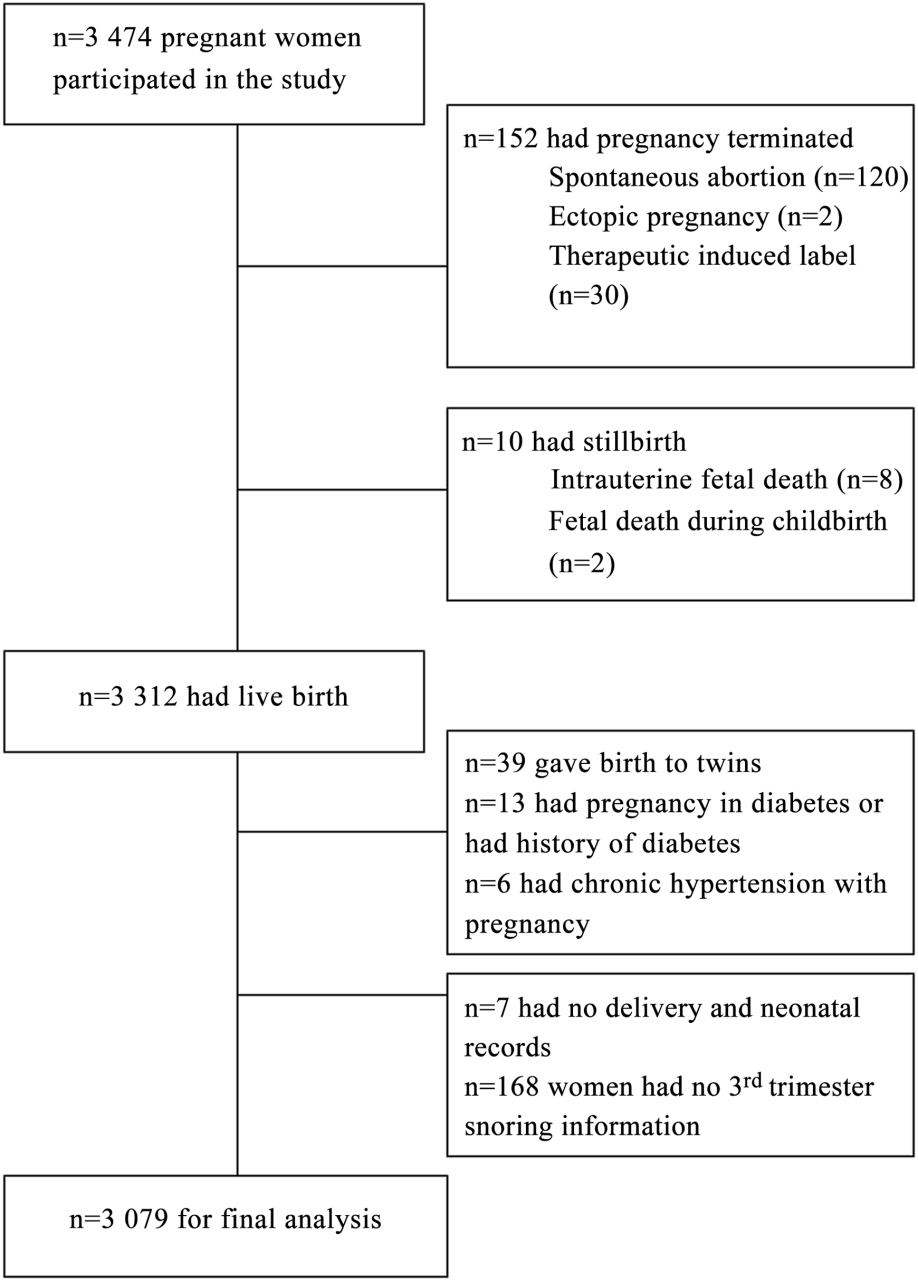
Recruitment flowchart.

A total of 3 474 pregnant women were invited to participate in the study. All of the participants answered the first questionnaire in the 1^st^ trimester. Among them, 152 women had pregnancy terminated, 10 women had stillbirth, 39 women gave birth twins, 7 women had no delivery and neonatal records, snoring information for 168 women in their third trimester were missing, 13 women were pregnancy complicated with diabetes or had a history of diabetes and 6 women had chronic hypertension complicating pregnancy were excluded, so the sample for this analysis is 3 079 (Fig 1). Overall, all participants had bed partners, and only 0.2% of partners complained about the snoring when women classified themselves as non-snorers. This didn’t change the associations with outcomes, so the self-reported answers were reliable and valid to a certain degree.

Overall, 16.6% (n = 511) pregnant women were habitual snorers, where 11.7% (n = 361) of them were pregnancy onset snorers, 4.9% (n = 150) were chronic snorers, and 83.4% of participants were non-snorers. Table 1 shows the baseline characteristics of pregnant women grouped by snoring status. There was a significant difference among pregnancy onset snorers, chronic snorers and non-snorers in maternal age, pre-pregnancy BMI, educational level, gravidity, smoking and only child or not (Table 1).

**Table 1:** Comparison of baseline characteristics in habitual snorers and non-snorers.

Variables	Habitual snorers (n = 511)		Non-snorers (n = 2 568)	*P*
	Pregnancy onset snorers (n = 361)	Chronic snorers (n = 150)		
Maternal age, y	26.50±3.54	27.11±4.56	25.98±3.53	<0.001
Pre-pregnancy BMI,kg/m^2^				<0.001
<18.5	44 (12.2)	14 (9.3)	524 (20.4)	
18.5–23.9	250 (69.3)	87 (58.0)	1799 (70.1)	
24.0–27.9	54 (15.0)	31 (20.7)	201 (7.8)	
≥28.0	13 (3.6)	18 (12.0)	44 (1.7)	
Gestational age at Enrollment, weeks	9.90±2.21	9.80±1.94	9.99±2.11	0.439
Ethnic,%				0.339
Han	358(99.2)	149(99.3)	2524(98.3)	
Other	3(0.80)	1(0.70)	44(1.70)	
Area of residence,%				0.266
Urban	294(81.4)	121(80.7)	2003(78.0)	
Rural	67(18.6)	29(19.3)	565(22.0)	
Educational level(years),%				0.041
≤9	77(21.3)	41(27.3)	493(19.2)	
10–15	178(49.3)	78(52.0)	1400(54.5)	
>15	106(29.4)	31(20.7)	675(26.3)	
Family per capita monthly income, (RMB)				0.220
<2500	100(27.7)	42(28.0)	659(25.7)	
2500–4000	147(40.7)	74(49.3)	1122(43.7)	
>4000	114(31.6)	34(22.7)	787(30.6)	
Gravidity,%				0.026
1	195(54.0)	68(45.3)	1444(56.2)	
2	100(27.7)	48(32.0)	741(28.9)	
≥3	66(18.3)	34(22.7)	383(14.9)	
Smoking				<0.001
Yes	16(4.4)	11(7.3)	57(2.2)	
No	345(95.6)	139(92.7)	2511(97.8)	
Only child or not				<0.001
Yes	151(41.8)	63(42.0)	821(32.0)	
No	210(58.2)	87(58.0)	1747(68.0)	

Continuous variables were shown as mean ± *SD*, categorical variables were shown as percentage.

Women who were not in our analysis were older (*t* = -3.15, *P* = 0.002) and had lower educational level (*χ*^*2*^ = 18.38, *P*<0.001) than the remaining women. There was no difference in area of residence (*χ*^*2*^ = 1.20, *P* = 0.273) family per capita monthly income (*χ*^*2*^ = 1.19, *P* = 0.756), or prevalence of habitual snoring before pregnancy (*χ*^*2*^ = 0.30, *P* = 0.581) between the sample in analysis and those excluded from analysis

Table 2 presents the incidence of adverse pregnancy outcomes from each group. Considering the types of snoring, we found that both pregnancy onset and chronic snorers were more likely to develop adverse pregnancy outcomes such as gestational diabetes, cesarean delivery and placental adhesion compared to non-snorers. Pregnancy onset snorers were more likely to delivery macrosomia and LGA infants compared to non-snorers. Conversely, in this study, maternal snoring did not increase the risk of gestational hypertension, preeclampsia or preterm birth (Table 2).

**Table 2:** Comparisons of the incidence of adverse pregnancy outcomes among groups.

Outcomes	Pregnancy onset snorers	Chronic snorers	Non-snorers
	(n = 361)	(n = 150)	(n = 2 568)
GDM	56(15.5)*	34(22.7)***	296(11.5)
Gestational hypertension	19(5.3)	10(6.7)	95(3.7)
preeclampsia	9(2.5)	4(2.7)	37(1.4)
Cesarean delivery	214(59.3)***	94(62.7)**	1244(48.4)
Preterm delivery	11(3.0)	10(6.7)	79(3.1)
LBW	7(1.9)	3(2.0)	47(1.8)
macrosomia	36(10.0)**	14(9.3)	161(6.3)
SGA	28(7.8)	8(5.3)	255(9.9)
LGA	91(25.2)***	32(21.3)	385(15.0)
Placental adhesion	21(5.8)**	13(8.7)***	69(2.7)

The reference group is non-snorers,

* means *P*<0.05,

** means *P*<0.01, and

***means *P*<0.001.

After controlling for potential covariates (pre-pregnancy BMI, maternal age, educational level, only child or not, gravidity, and smoking), logistic regressions showed that pregnancy onset snoring increased the risk of caesarean delivery (*RR* 1.37, 95%*CI* 1.09–1.73) macrosomia (*RR* 1.54, 95%*CI* 1.05–2.27) and LGA (*RR* 1.71, 95%*CI* 1.31–2.24). It’s chronic, but not pregnancy onset snoring was independently associated with GDM (*RR* 1.66, 95%*CI* 1.09–2.53). The risk of placental adhesion existed in both pregnancy onset and chronic snoring groups (*RR* 1.96, 95%*CI* 1.17–3.27 and *RR* 2.33, 95%*CI* 1.22–4.46, respectively) (Table 3).

**Table 3:** Regression analyses of maternal and newborn outcomes against snoring and other covariates.

Variable	Beta	*SE*	Adjusted *RR*	95% *CI*
GDM				
Pregnancy onset snorers	0.19	0.16	1.20	0.88–1.65
Chronic snorers	0.51	0.21	1.66	1.09–2.53
Cesarean delivery				
Pregnancy onset snorers	0.32	0.12	1.37	1.09–1.73
Chronic snorers	0.28	0.18	1.32	0.93–1.89
Macrosomia				
Pregnancy onset snorers	0.43	0.20	1.54	1.05–2.27
Chronic snorers	0.21	0.30	1.23	0.68–2.23
LGA				
Pregnancy onset snorers	0.54	0.14	1.71	1.31–2.24
Chronic snorers	0.17	0.22	1.18	0.77–1.80
Placental adhesion				
Pregnancy-onset snorers	0.67	0.26	1.96	1.17–3.27
Chronic snorers	0.85	0.33	2.33	1.22–4.46

Covariates in model: maternal age, educational level, gravidity, only child or not, maternal smoking, and pre-pregnancy BMI.

Since pre-pregnancy BMI was significantly associated with maternal snoring (Table 1). To further examine the associations of snoring status and pregnancy outcomes. Participants were shown as snorers (chronic snorers and pregnancy onset snorers were merged into snorers) vs non-snorers, then they were divided into four groups according to pre-pregnancy BMI. They were lean non-snorers (non-snorers with BMI < 24.0 kg/m^2^, n = 2 323), lean snorers (snorers with BMI < 24.0 kg/m^2^, n = 395), overweight/obesity non-snorers (non-snorers with BMI≥ 24.0, n = 245), and overweight/obesity snorers (snorers with BMI≥ 24.0, n = 116). Lean non-snorers were regarded as the reference group when conducting logistic regressions. After controlling for the same confounders conducted in Table 3, which may be related to adverse pregnancy outcomes, Table 4 presents the *RR* values and 95%*CI* of pre-pregnancy BMI with or without snoring in each regression model for adverse pregnancy outcomes.

**Table 4:** Adjusted *RR* and 95%*CI* of pregnancy outcomes by pre-pregnancy BMI and snoring status.

Variable	Beta	SE	Adjusted *RR*	95% *CI*
GDM				
Lean snorers	0.50	0.15	1.65	1.22–2.23
Overweight/obese non-snorers	0.98	0.17	2.68	1.94–3.70
Overweight/obese snorers	0.83	0.24	2.29	1.44–3.64
Gestational hypertension				
Lean snorers	0.32	0.33	1.38	0.73–2.63
Overweight/obese non-snorers	1.43	0.28	4.19	2.41–7.29
Overweight/obese snorers	1.44	0.39	4.24	1.98–9.11
Preeclampsia				
Lean snorers	0.15	0.45	1.16	0.48–2.82
Overweight/obese non-snorers	0.93	0.43	2.54	1.09–5.94
Overweight/obese snorers	1.67	0.44	5.29	2.22–12.62
Cesarean delivery				
Lean snorers	0.32	0.11	1.37	1.10–1.71
Overweight/obese non-snorers	0.49	0.14	1.63	1.24–2.15
Overweight/obese snorers	0.95	0.22	2.58	1.69–3.92
Preterm birth				
Lean snorers	0.11	0.31	1.12	0.61–2.06
Overweight/obese non-snorers	0.63	0.31	1.87	1.03–3.41
Overweight/obese snorers	0.83	0.39	2.30	1.07–4.95
Macrosomia				
Lean snorers	0.47	0.20	1.61	1.09–2.37
Overweight/obese non-snorers	0.60	0.23	1.83	1.16–2.87
Overweight/obese snorers	0.82	0.30	2.27	1.25–4.11
LGA				
Lean snorers	0.50	0.14	1.65	1.26–2.16
Overweight/obese non-snorers	0.53	0.17	1.70	1.23–2.35
Overweight/obese snorers	0.86	0.22	2.35	1.54–3.60
Placental adhesion				
Lean snorers	0.85	0.26	2.33	1.40–3.88
Overweight/obese non-snorers	0.86	0.31	2.36	1.30–4.30
Overweight/obese snorers	1.33	0.35	3.80	1.91–7.56

Covariates in model: maternal age, educational level, gravidity, only child or not, and maternal smoking.

Compared to lean non-snorers, both lean and overweight/obese snorers were at high risk for GDM (*RR* 1.65, 95%*CI* 1.22–2.23, and *RR* 2.29, 95%*CI* 1.44–3.64, respectively), cesarean delivery (*RR* 1.37, 95%*CI* 1.10–1.71, and *RR* 2.58, 95%*CI* 1.69–3.92, respectively), placental adhesion (*RR* 2.33, 95%*CI* 1.40–3.88, and *RR* 3.80, 95%*CI* 1.91–7.56, respectively), macrosomia (*RR* 1.61, 95%*CI* 1.09–2.37 and *RR* 2.27, 95%*CI* 1.25–4.11, respectively) and LGA (*RR* 1.65, 95%*CI* 1.26–2.16, and *RR* 2.35, 95%*CI* 1.54–3.60, respectively). In models of gestational hypertension, preeclampsia, and preterm birth, overweight/obese snorers had significantly increased *RR*s (*RR* 4.24, 95%*CI* 1.98–9.11, *RR* 5.29, 95%*CI* 2.22–12.62, and *RR* 2.30, 95%*CI* 1.07–4.95, respectively) than lean non-snorers. Lean snorers were not at increased risk of gestational hypertension, preeclampsia, or preterm birth (*RR* 1.38, 95%*CI* 0.73–2.63, *RR* 1.16, 95%*CI* 0.48–2.82, and *RR* 1.12, 95%*CI* 0.61–2.06, respectively). In these models, overweight/obese non-snorers also increased the adverse outcomes (*RR* 2.68, 95%*CI* 1.94–3.70 for gestational diabetes, *RR* 1.63, 95%*CI* 1.24–2.15 for cesarean delivery, *RR* 2.36, 95%*CI* 1.30–4.30 for placental adhesion, *RR* 1.83, 95%*CI* 1.16–2.87 for macrosomia, *RR* 1.70, 95%*CI* 1.23–2.35 for LGA, *RR* 4.19, 95%*CI* 2.41–7.29 for gestational hypertension, *RR* 2.54, 95%*CI* 1.09–5.94 for preeclampsia, and *RR* 1.90, 95%*CI* 1.05–3.46 for preterm birth). Moreover, in all these models, overweight/obese snorers had the highest *RR*s than other groups except for GDM.

Only one woman reported witnessed apneas before pregnancy, and 3.7% (n = 115) of pregnant women reported the symptom during pregnancy. Logistic regressions showed that witnessed apneas were only associated with preterm birth (*RR*, 2.55, 95%*CI*, 1.24–5.22) after controlling for maternal age, educational level, gravidity and maternal smoking.

## Discussion

This is the first prospective cohort study with a large sample to investigate the associations between maternal snoring status and pregnancy outcomes in China. Our findings indicate that both pregnancy onset and chronic snoring are predictors of placental adhesion, chronic snorers are independently associated with GDM, and pregnancy onset snorers are more likely to have caesarean delivery, macrosomia and LGA infants. However, neither pregnancy onset nor chronic snorers are related to gestational hypertension, preeclampsia or preterm birth. Additionally, regardless of the type of snoring, it is demonstrated that being overweight or obese before pregnancy plays an important role in mediating the effect of maternal snoring on adverse pregnancy outcomes after grouping.

Previous studies have reported that snoring disturbed 6.7%-9.0% of women, but the rate seemed to increase to 24.0%-28.0% when they were pregnant [1,13,31]. One prospective study objectively measured SDB symptoms by using polysomnography (PSG) reported that the frequency of SDB peaked in the third trimester [22]. Our study reported a lower prevalence of habitual snoring both before and during pregnancy than existing literature, but it revealed a progressive increase from the first to the third trimester of pregnancy as previously reported [2,4,13].Moreover, the frequency of snoring increases during pregnancy is consistent with the theory that upper airway resistance increased in the 2^nd^ and 3^rd^ trimester [32].

### Snoring and adverse maternal outcomes

To examine the relationship between frequent snoring (snoring≥3 nights per week) and GDM, Facco et al found that pregnant women with frequent snoring had evaluated oral glucose tolerance values and higher incidence of GDM than non-snorers despite after controlling for potential confounders [33]. In addition, researchers have demonstrated that women who snored were nearly 2 times more likely to have GDM, and overweight snorers were 5.9 times higher to have GDM compared to lean snorers [34]. Subsequent meta-analysis further confirmed the conclusion [15]. The mechanism underlying snoring and GDM remains unclear. In non-pregnant populations, SDB was considered to link with insulin resistance and impaired glucose tolerance, these pathogenesis may also be applied to pregnant women with sleep disturbance [14,35,36].

In this study, we have a novel finding that both pregnancy onset and chronic snoring were associated with significantly higher risks of placental adhesion. While there is no direct evidence showing a relationship between snoring and placental adhesion so far. Intermittent hypoxia caused by snoring may have some mediating effect. Studies suggested that hypoxia during pregnancy could cause vascular changes and affect trophoblast cell adhesion, proliferation, invasion and apoptosis, which had adverse effects on placental function and might lead to placental adhesion [37–39]. Contrary to previous studies, we did not find any association between snoring and gestational hypertension and preeclampsia. Existing literature has shown controversial conclusions on snoring and gestational hypertension disorders in different countries [13,40,41]. These suggest that country and racial differences may act as potential mediators. In addition, the lower frequency of pre-pregnancy overweight/obesity may partly explain the discrepancy, since pre-pregnancy BMI is significantly associated with both snoring and gestational hypertension and preeclampsia, whereas the prevalence of overweight and obesity in China is much lower than Western and European countries. Also, the differences of age and educational level between the sample in analysis and those excluded from analysis in this study may be associated with the results. Therefore, the roles of snoring in placental adhesion, gestational hypertension and preeclampsia merit further investigation.

Our findings show that women with pregnancy onset snoring are more likely to undergo caesarean delivery. The main reason for this situation may be due to sleep deprivation and fatigue caused by snoring. Studies showed that women with habitual snoring were nearly 2 times more likely to have unplanned caesarean delivery after adjusting for other confounders [12,18,42]. Furthermore, we also find the risk of caesarean delivery in overweight/obese snorers is almost twice than lean snorers, this suggests that being overweight or obese before pregnancy may exacerbate the outcome caused by snoring, and prior studies have drawn a conclusion that habitual snoring women had higher BMI, and more likely to undergo caesarean delivery [43–45].

### Snoring and adverse neonatal outcomes

Despite mounting researches linking maternal snoring with adverse maternal outcomes, evidence on maternal snoring and neonatal outcomes is conflicted. Though some studies reported maternal SDB increased the risk of SGA or intrauterine growth restriction [18,21], other studies failed to support the findings [12,46]. A prospective study evaluated OSA through Berlin Questionnaire showed that neonatal of OSA mothers were more likely to have higher birth weight than the matched group [41]. The underlying mechanism of maternal SDB on neonatal outcomes seems complex, as the consequence of maternal SDB includes both GDM and gestational hypertension, which may have the opposite effect to the fetus [47]. Our finding merits further confirmation and discussion for future researchers.

The strengths of this study include prospective design, large sample size, high response rates, controlling for other contributing factors, a population representative medical centre, and the Chinese ethnicity of the population. There have been numerous studies on maternal SDB and adverse pregnancy outcomes in the West but data are lacking from Asia. Our study has reported the similar findings to the Western world. Conducting this study in China also allows examination of the associations between snoring and adverse outcomes without the major confounder of overweight or obesity, since pre-pregnancy overweight/obesity is a stronger confounder for many adverse pregnancy outcomes, whereas the prevalence of pre-pregnancy overweight/obesity is low in our study (11.7%). These are additional information this study provides beyond the literature that have already been published. In addition, we investigated the pre-pregnancy habitual snoring from the first trimester, which can reduce recall bias of our study participants.

One major limitation of our study is the lack of an objective method such as overnight polysomnogram (PSG) to measure habitual snoring. However, the aim of this study was to validate the use of snoring directly predicting adverse pregnancy outcomes, and existing literature has confirmed that self-reported snoring was strongly associated with the PSG-derived sleep apnea hypopnea index [48–50]. We also used self-reported weight and height data to calculate pre-pregnancy BMI, which may increase measurement bias, and pre-pregnancy overweight and obesity possesses a lower proportion than the West reported, this could be a large limitation to examine for adverse pregnancy outcomes, While in this study, though pre-pregnancy overweight and obesity accounts for a low percentage, it acts as an important role in mediating both adverse maternal and neonatal outcomes, when snoring status was stratified by pre-pregnancy BMI, further significant associations between snoring and adverse outcomes were found. Moreover, the low percentage of pre-pregnancy overweight/obesity may result in the lower prevalence of snoring in pregnancy in this study than previously published literature, as studies suggested that overweight and obese pregnant women were at higher risk of sleep-disordered breathing than their lean weight counterparts [24,25]. Another weakness is that only pregnant women whose obstetrics visit was at the MCH Care Center were included, even though the center contains about 80% of pregnant women. This may likely to increase the selection bias, and the results are difficult to promote to the whole country.

All participants in the study answered the first questionnaire, while there were 11.4% (n = 395) of pregnant women were not included in the final analysis sample due to pregnancy termination before delivery, stillbirth, and the third trimester snoring information missing et al, which might increase non-response bias, however, drop-out rate analysis indicated no difference of prevalence of habitual snoring before pregnancy between the sample in analysis and those excluded from analysis. (*P* = 0.581).

In conclusion, our study indicates that women are more likely to experience habitual snoring during pregnancy than non-pregnant population. We also find that maternal snoring during pregnancy is independently associated with adverse pregnancy outcomes, such as GDM, caesarean delivery, placental adhesion, macrosomia and LGA. Being overweight or obese before pregnancy was found to play an important role in mediating maternal snoring and adverse pregnancy outcomes. These findings may have important clinical implications. The mechanisms underlying SDB need further study so that Obstetricians can take intervention timely to improve maternal-fetal health.
